# The American and Colonial Journals: Anatomy and Physiology

**Published:** 1840-04

**Authors:** 


### II. THE AMERICAN AND COLONIAL JOURNALS.

### ANATOMY AND PHYSIOLOGY.

*Report of Experiments on the Action of the Heart.* By C. W. Pennock, M.D., Physician to the Philadelphia Hospital, and E. M. Moore, M.D, late Resident Physician to the Frankford Asylum.

[These experiments, fifteen in number, were performed on sheep, calves, and one horse; we have only room for the conclusions deduced from them by the experimenters.]

1st. The impulse is synchronous with and caused by the ventricular contraction, and when felt externally, arises from the striking of the apex of the heart against the thorax.

2d. The expulsion of the blood from the ventricles is effected by an approximation of the sides of the heart only, and not by a contraction of the apex towards the base; during the systole the heart performs a spiral movement, and becomes elongated. (Experiments 6th, 10th, and 11th.)

3d. The ventricle contracts and the auricle dilates at the same time, occupying about one half of the whole time required for contraction, diastole, and repose. Immediately at the termination of the systole of the ventricle, its diastole succeeds, occupying about one fourth of the whole time, synchronous with which the auricle diminishes, by emptying a portion of its blood in the ventricle, unaccompanied with muscular contraction. The remaining fourth is devoted to the repose of the ventricles, near the termination of which the auricle contracts actively, with a short, quick motion, thus distending the ventricles with an additional quantity of blood: this motion is propagated immediately to the ventricles, and their systole takes place, rendering their contractions almost continuous. (Experiments 15 and 16.)

4th. From the termination of their diastole to the commencement of their systole, the ventricles are in a state of perfect repose, their cavities remaining full, but not distended, while those of the auricles are partially so, during the whole time.

5th. The sounds are produced by the motions of the heart or its contents, and not by striking against the thorax, as proved in all the experiments; being much louder when the stethoscope was applied directly to the heart than when to the chest, or with the lungs interposed.

6th. The sounds are more distinct when the muscle is thin and contracts quickly. Hence the clear flapping character of the first sound over the right ventricle, as compared with the left.

7th. The first sound, the impulse, and the ventricular systole, are synchronous. This sound may be a combination of that caused by the contraction of the auricles, the flapping of the auriculo-ventricular valves, the rush of blood from the ventricles, and the sound of muscular contraction. From experiments 3d, 4th, 6th, and 10th, when the heart was removed from the body, the ventricles cut open and emptied of their contents, the auriculo-ventricular valves elevated, and a sound, resembling the first, still heard, it may be chiefly attributed to the muscular contraction. That these valves aid but slightly in its production may also be inferred from experiment 16.

8th. The second sound is caused exclusively by the closure of the semi-lunar valves, from the tendency to regurgitate of the arterial column of blood upon them. This is proved by the greater intensity of this sound over the aorta than elsewhere, the blood having a strong tendency to return through the valvular opening; by the greater feebleness of the sound over the pulmonary artery, which is short, and soon distributes its blood through the lungs, thus producing but slight impulse upon the valves in the attempt to regurgitate; by the disappearance of the sound, when the heart becomes congested and contracts feebly; and, finally, on account of its entire extinction when the valve of the aorta was elevated.

9th. The second sound is synchronous with the diastole of the ventricle.

From these experiments it will be seen that our conclusions coincide very nearly with those of the British physiologists—the correctness of whose results, when compared with those of the French, may be mainly attributed to the use of larger animals. From our observations, calves, of from four to eight weeks old, are decidedly preferable to other quadrupeds for these investigations. The tenacity of life of calves of this age is greater than in older animals, whilst the cardiac pulsations are slower and more forcible than they are in the younger. The heart of this animal, too, is of large size, and the introduction of hooks for the elevation of the valves is readily effected.

Remarks [by the Editors of the Medical Examiner.] It will be perceived by a reference to the experiments, that besides confirming several points already set forth by the British experimenters, the experiments of Dr. Pennock have developed some new facts. These are the existence of an auricular sound, faint indeed, and to a great degree merged in that of the ventricles, but still real. Indeed, when the fact of real muscular contraction of the auricles is proved, we know from analogical reasoning that sound, which is the necessary consequence of muscular contraction, must be produced. The active muscular contraction of the auricles has been doubted by some physiologists, we hardly know why, for the evident muscular fibres of the auricles are certainly designed for no other purpose than other muscles, that is, contraction. The experiments of Drs. Pennock and Moore have set this question also at rest, and proved satisfactorily to all present, that the muscular fibres of the auricles were in action during their systole.

There are two other points ascertained positively by these observers, from direct experiment, which we believe were not noted in previous observations of the kind. These are—1st, that the ventricular sound on the right is much sharper and clearer than on the left, which is more dull and prolonged; 2d, that the second or valvular sound ceases by the congestion of the ventricles, ceasing first on the right side. With both of these facts we have long been familiar, from our observations of the sounds of the heart in the living body, both in the healthy and diseased condition of this organ; and it is not a little gratifying to us to find that the clinical observations which we have often made and pointed out to our pupils have been abundantly confirmed by the sure test of direct experiment. This observation throws light upon a large number of pathological facts, which are often with difficulty understood without knowing the effect of congestion upon the heart.

The last point of interest which has been for the first time explicitly stated, is the less degree of loudness of the second sound over the pulmonary artery and valves than over the aorta. This probably arises from the pulmonary valves being so much weaker and thinner.

*American Medical Examiner. Nov. 2, 1839.*

*On the Sedative Effects of Ergot (Experiments with the Powder).* By J. B. COTTMAN, M.D., Resident Physician of the Philadelphia Hospital.

THE subjects of these experiments were strong healthy men, but lunatics. A diminution in the frequency of the pulse, and, in several, of the volume, were the only phenomena observed, except in three cases; in these it produced slight nausea, but no vomiting; in two others, to whom it was given the day before, violent emesis was brought on in the course of one hour. When administered in half-dram doses, it either produced nausea—and consequently sedation—or diminished the frequency of the pulse, without nauseating, in the course of twenty-five or thirty minutes; but, when given in scruple doses, it appeared to excite rather than diminish the pulse in that time; in fifteen or twenty minutes, however, the pulse lost a few beats in the minute, but not to the same extent as when given in larger doses: in several cases it produced little or no effect when given in the smaller doses. No other cause operated in producing this result, unless we presume that the fact of giving the men medicine when they were in health excited them; and as I examined the pulse in every case just before giving the medicine, and forty or forty-five minutes afterwards, the excitement might have passed off, and, consequently, the pulse be diminished in frequency; this, indeed, is not unlikely, as the pulse in several cases was much above the natural standard.

It is necessary, I need scarcely say, to be very cautious in drawing conclusions with regard to the medical properties of any drug from a limited number of experiments, but I think we may safely infer, that the ergot has a power of depressing the pulse when given in large doses, but may question very much its sedative agency in small doses. The following is a list of the cases. The age of each individual is affixed to his name:

| Names. | Age. | Pulse at time of administration. | Pulse forty-five minutes afterwards. |
|---|---|---|---|
| Daley . | 62 | 72 | 60 |
| Young . | 20 | 80 | 64 |
| Dougherty | 30 | 88 | 70 |
| Hews . | 20 | 70 | 60 |
| Daughney | 45 | 104 | 80 |
| M'Cally . | 35 | 84 | 68 |
| Buck . | 35 | 80 | 66 |
| M'Carit . | 30 | 60 | 50 |
| Thompson | 50 | 84 | 72 |
| Stewart . | 40 | 76 | 70 |
| Campbell | 29 | 68 | 60 |
| M'Donald | 22 | 120 | 106 |
| Daley . | 62 | 68 | 60 |
| O'Brien . | 33 | 76 | 60 |
| *Cases in which Scruple Doses were given.* | | | |
| Bush . | 60 | 84 | 78 |
| Leonard . | 31 | 92 | 86 |
| Leaman . | 19 | 88 | 84 |
| Caryer . | 30 | 72 | 70 |
| Nealy . | 40 | 80 | 76 |
| Campbell | 30 | 64 | 58 |
| Thompson | 40 | 92 | 88 |
| Fagan . | 30 | 84 | 80 |
| M'Nice . | 40 | 68 | 62 |
| Stomeyer | 30 | 80 | 78 |

*American Medical Intelligencer.* No. xi

